# Comparative Effectiveness of Techniques in Targeted Prostate Biopsy

**DOI:** 10.3390/cancers13061449

**Published:** 2021-03-22

**Authors:** Dordaneh Sugano, Masatomo Kaneko, Wesley Yip, Amir H. Lebastchi, Giovanni E. Cacciamani, Andre Luis Abreu

**Affiliations:** 1 USC Institute of Urology and Catherine & Joseph Aresty Department of Urology, Center for Image-Guided and Focal Therapy for Prostate Cancer, Keck School of Medicine, University of Southern California, Los Angeles, CA 90089, USA; dordaneh.sugano@med.usc.edu (D.S.); masatomo.kaneko@med.usc.edu (M.K.); wesleyyi@med.usc.edu (W.Y.); Amir.Lebastchi@med.usc.edu (A.H.L.); Giovanni.Cacciamani@med.usc.edu (G.E.C.); 2 Department of Urology, Graduate School of Medical Science, Kyoto Prefectural University of Medicine, Kyoto 602-8566, Japan

**Keywords:** prostate cancer, magnetic resonance imaging, fusion biopsy, systematic biopsy

## Abstract

**Simple Summary:**

Prostate cancer is one of the most common cancers in men. Traditionally, prostate cancer is diagnosed via transrectal ultrasound-guided prostate biopsy, using a systematic random template. Using multiparametric magnetic resonance imaging, lesions suspicious for prostate cancer can be identified, and subsequently targeted on biopsy, allowing for increased diagnostic accuracy. This article reviewed the current literature surrounding various types of targeted biopsy, such as transperineal biopsy, allowing for comparison not only between targeted biopsy and systematic biopsy, but also between different varieties of targeted biopsy.

**Abstract:**

In this review, we evaluated literature regarding different modalities for multiparametric magnetic resonance imaging (mpMRI) and mpMRI-targeted biopsy (TB) for the detection of prostate cancer (PCa). We identified studies evaluating systematic biopsy (SB) and TB in the same patient, thereby allowing each patient to serve as their own control. Although the evidence supports the accuracy of TB, there is still a proportion of clinically significant PCa (csPCa) that is detected only in SB, indicating the importance of maintaining SB in the diagnostic pathway, albeit with additional cost and morbidity. There is a growing subset of data which supports the role of TB alone, which may allow for increased efficiency and decreased complications. We also compared the literature on transrectal (TR) vs. transperineal (TP) TB. Although further high-level evidence is necessary, current evidence supports similar csPCa detection rate for both approaches. We also evaluated various TB techniques such as cognitive fusion biopsy (COG-TB) and in-bore biopsy (IB-TB). COG-TB has comparable detection rates to software fusion, but is operator-dependent and may have reduced accuracy for smaller lesions. IB-TB may allow for greater precision as lesions are directly targeted; however, this is costly and time-consuming, and does not account for MRI-invisible lesions.

## 1. Introduction

With the advent of advanced imaging techniques and image-guided biopsy for the detection of prostate cancer (PCa), there have been innumerable series evaluating the efficacy of both multiparametric magnetic resonance imaging (mpMRI) and mpMRI-targeted biopsy (TB). In this review, we discuss the efficacy of TB compared to systematic biopsy (SB), as well as the various methods and comparative effectiveness involved with different TB techniques.

The main three approaches to TB include cognitive fusion (COG-TB), software-based fusion (FUS-TB), and in-bore or in-gantry TB (IB-TB). COG-TB involves an operator cognitively evaluating previously obtained mpMRI images and using anatomic landmarks to target suspicious lesions on real-time transrectal ultrasound (TRUS). However, this strategy relies heavily on operator skill [1]. FUS-TB utilizes software to overlay previously obtained mpMRI images on real-time TRUS images, prior to sample acquisition. IB-TB is performed in the MRI suite with real-time MRI guidance [2].

## 2. Systematic Versus Targeted Biopsy

There is a large body of data, including a recent randomized trial, demonstrating superior clinically significant PCa (csPCa) detection for MRI-informed TB compared to SB [3] (Table 1). Recently, Ahdoot et al. evaluated over 2000 men with MRI-visible lesions who underwent both transrectal FUS-TB and SB [4]. Detection rates of csPCa defined as grade group (GG) ≥ 2 were 31% in the SB cohort and 37.8% in the TB cohort. With statistical analysis, detection rates were found to be significantly higher for TB for GG 3–5, and significantly lower for GG 1. They calculated a 21.8% rate of upgrading on GG when TB was added to SB. However, TB alone missed GG ≥ 2 PCa in 5.8% of patients and GG ≥ 3 in 1.9% of patients. Rates of upgrading on prostatectomy specimens were found to be significantly higher for SB 41.6% (16.8% csPCa upgrading) compared to TB 30.9% (8.7% csPCa upgrading). Combined SB + TB upgrading rates were 14.4% and 3.5%, respectively, for overall upgrading and csPCa upgrading. The low incidence of csPCa detected in SB alone, as well as the low risk of upgrading from TB alone, provided further support for the efficacy of TB. An earlier National Cancer Institute study evaluated 1003 patients who also underwent both FUS-TB and SB [5]. They found that while TB and SB diagnosed comparable rates of PCa (461 vs. 469 cases), TB diagnosed 30% more high-risk (GG ≥ 3) PCa. In analysis of CDR by the Gleason score, they reported that 263 of the 1003 patients (26%) had GG ≥ 2 PCa diagnosed on SB, vs. 314 cases (31%) on TB. The increased csPCa detection rate seen in the 2020 study may reflect the learning curve for TB [6], as well as advances in technology. Notably, both cohorts have a higher proportion of prior-negative biopsy patients (43% in 2015, and 41.5% in 2020), which both studies note as a limitation.

Filson et al. also evaluated a large cohort (including men with suspicion of PCa as well as men with known PCa considering active surveillance) undergoing both FUS-TB and SB [7]. For patients with a region of interest (ROI) grade ≥ 3 on an internal scoring system for mpMRI, they determined that TB alone identified csPCa (GG ≥ 2) in 229/825 cases (28%), vs. 199 (24%) for SB, and 289 (35%) for SB + TB. They determined that the combined approach identified a significantly higher proportion of csPCa compared to both SB and TB alone (*p* < 0.001 for both). They determined that the number needed to biopsy with the combined approach to identify one additional case of csPCa was 14, and that for each diagnosis of csPCa, combined biopsy would identify one additional case of clinically insignificant PCa (ciPCa).

## 3. Systematic Versus Targeted Biopsy in the Biopsy-Naïve Setting

Maxeiner et al. also compared FUS-TB to SB (10 core), specifically in biopsy-naïve patients in their retrospective analysis of 318 patients with the Prostate Imaging Reporting and Data System (PIRADS) ≥ 3 lesions [8]. They found csPCa in 51% of patients (GG ≥ 3 or maximum cancer core length of ≥6 mm) on TB, 46% on SB, and 61% on combined biopsy. Gleason upgrading on SB compared to results on TB was seen in 32% of patients, of which 9% were upgraded to csPCa, compared to 26% of patients with upgrading on TB compared to SB (14% upgraded to csPCa). They showed that combined biopsy detected significantly higher rates of, in particular, GG ≥ 4 compared to both SB and TB (*p* < 0.001). Similarly, in their prospective study, Pokorny et al. analyzed 223 biopsy-naïve patients, of whom the 143 with PIRADS ≥ 3 lesions underwent both SB and TB (modality unclear), while MRI-negative patients underwent SB only [9]. Overall CDR for SB was 56.5%, with a 62.7% intermediate/high risk (high volume GG2, GG ≥ 3), compared to CDR 69.7% with a 93.9% intermediate/high risk for TB. On combined SB + TB, CDR was 64%, of which 76% were intermediate/high risk. Of note in this study is the relatively high proportion of intermediate/high risk patients diagnosed on TB alone, although it is difficult to determine the exact modality of biopsy.

The 4-M trial also evaluated biopsy-naïve patients (IB-TB vs. SB) [10]. They obtained mpMRI in all patients, and in the subset with PIRADS ≥ 3 lesions, they performed IB-TB followed by SB (MRI pathway). MRI-negative patients underwent SB alone (TRUS guided biopsy [TRUSGB] pathway). In patients with PIRADS 3–5 lesions, MRI-TB identified csPCa (GG ≥ 2) in 50% of patients, while a combination of MRI-TB and SB identified csPCa in 57%. Overall, the TRUSGB pathway identified csPCa in 23%, compared to 25% in the MRI pathway. They reported that MRI-TB alone underdetected 9% of csCPa vs. 2% in the combined approach. When comparing the sensitivity of the combined approach to SB, they determined that there was no significant difference in the detection of csPCa. The relative similarity of the MRI and TRUSGB pathways may be influenced by the biopsy-naïve nature of the population. As this multi-institutional study included several non-university hospitals, these results may speak to the role of mpMRI and TB in a community setting.

The MRI-FIRST study, unlike similar studies, found comparable CDR for both SB and TB [11]. In this prospective study, biopsy-naïve patients underwent mpMRI, with subsequent SB with up to 2 cores targeting hypoechoic lesions. Patients with Likert score ≥ 3 lesions underwent TB in accordance with institutional standard of care: COG-TB, FUS-TB, or COG-TB combined with a contrast-enhanced ultrasound. GG ≥ 2 PCa was diagnosed in 32.3% of TB alone cases, 29.9% of SB alone cases, and 37.5% of combined cases. Although there was no significant difference between SB and TB in the detection of GG ≥ 2 PCa, TB was found to detect a significantly higher rate of GG ≥ 3 PCa (19.9% vs. 15.1%, *p* = 0.0095). Conversely, SB detected a significantly higher proportion of ciPCa (20% vs. 5.8%, *p* < 0.0001). The authors determined that SB alone would miss 7.6% of csPCa, while mpMRI would miss 5.2%. It is difficult to calculate how the additional variable of ultrasound-targeted biopsy cores affected these results in comparison to other studies.

In summary, several studies evaluating SB and TB in the same patient showed the importance of maintaining SB [12]. SB improves detection of csPCa when combined with TB, and information obtained from SB may contribute to prostate mapping and treatment planning, such as for partial gland ablation [13]. However, SB increases the detection of ciPCa, and may therefore lead to overtreatment of PCa. Furthermore, SB increases complication rates, the burden of biopsies on pathologists, and potentially increases costs [14,15]. As the technology improves, future studies will determine whether patients with suspicious MRI (PIRADS 3–5) can omit SB without compromising csPCa detection.

## 4. Systematic Versus Targeted Biopsy Using the Transperineal Approach

**Figure 1:**
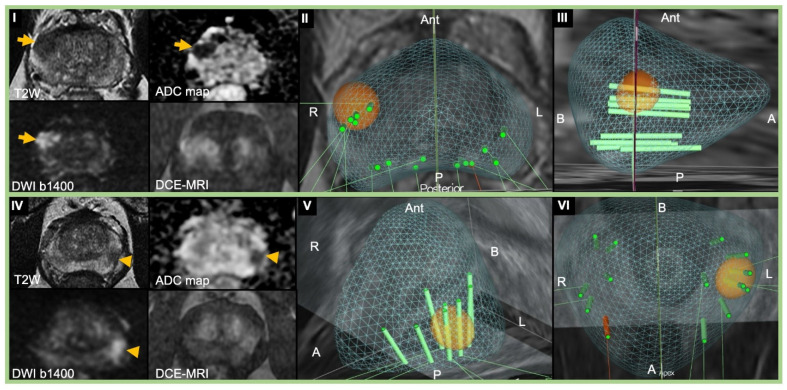
Representative images of 3D-TRUS/MRI fusion-guided transperineal and transrectal prostate biopsy. (**I**–**III**) A 75-year-old man with PSA 9.48 ng/mL who underwent 3D-TRUS/MRI fusion-guided TP prostate biopsy under local anesthesia with Gleason Grade Group 5 prostatic adenocarcinoma detected on target biopsy without any complications. (**I**) Pre-biopsy multiparametric MRI showing a 1.2 cm PI-RADS 4 lesion (arrow) in the right mid-anterior peripheral zone. The lesion was moderately hypointense on T2W, focal markedly hypointense on the ADC map, markedly hyperintense on high b-value DWI, and showed focal early enhancement on DCE. (**II**) Transverse and (**III**) right sagittal view of 3D-TRUS/MRI fusion-guided TP prostate biopsy (green cores) cartography. Blue mesh is the contour of the prostate. Target (orange sphere) was assigned on right mid-anterior peripheral zone PI-RADS 4. (**IV**–**VI**) A 62-year-old man with PSA 5.98 ng/mL who underwent 3D-TRUS/MRI fusion-guided TR prostate biopsy under local anesthesia with Gleason Grade Group 3 prostatic adenocarcinoma detected on target biopsy without any complications. (**IV**) Pre-biopsy multiparametric MRI showing 0.9 cm PI-RADS 4 lesion (arrowhead) in the left mid posterior peripheral zone. The round-shaped lesion was moderately hypointense on T2W, focal markedly hypointense on the ADC map, markedly hyperintense on high b-value DWI, and showed focal early enhancement on DCE. The segmentation is the same as TP biopsy. (**V**) Left sagittal view and (**VI**) coronal view of 3D-TRUS/MRI fusion-guided TR prostate biopsy (green cores) cartography. Target was assigned on left posterior apex peripheral zone. T2W, T2 weighted; ADC, apparent diffusion coefficient; DWI, diffusion-weighted imaging; DCE, dynamic contrast-enhanced; A, apex; B, base; R, right; L, left; Ant, anterior; P, posterior; PSA, prostate-specific antigen; MRI, magnetic resonance image; PI-RADS, Prostate Imaging Reporting and Data System; 3D, three-dimension; TRUS, transrectal ultrasound; TP, transperineal; TR, transrectal.

The transperineal prostate biopsy (TP) approach has been gaining in popularity (Figure 1), partly given concerns with the transrectal approach (TR) for increasing usage of prophylactic broad-spectrum antibiotics, rising bacterial resistance to antibiotics, higher risk of infection, and its inherent risk of rectal bleeding [16]. Although it has been established that the CDR of SB with TP or TR approaches is comparable, it is not yet well studied which approach is superior to the other with regard to FUS-TB [17].

Loy et al. compared the diagnostic accuracy of TP vs. TR FUS-TB in the detection of csPCa in their systematic review and meta-analysis [16]. According to the meta-analysis, the pooled sensitivity of TR and TP approaches were 0.81 and 0.80, respectively. The pooled specificity of TR and TP approaches were 0.99 and 0.95, respectively. The area under the receiver operator curve of TR and TP approaches was 0.91 and 0.88, respectively. Although these diagnostic performances were similar, the positive likelihood ratios and diagnostic odds ratios for the TR approach were higher than the TP approach.

Pepe et al. evaluated the detection rate for csPCa of TP vs. TR FUS-TB in their prospective analysis of 150 repeat biopsy patients [18]. They showed that the detection rate for csPCa using TP FUS-TB was higher than TR (89.1% vs. 78.1%). This superiority may be due to the higher detection rate of PCa in the anterior zone than TR (86.7% vs. 46.7%; *p* = 0.0001). Furthermore, the TP approach was able to detect csPCa in smaller mpMRI lesions than TR (8 vs. 12 mm). TP FUS-TB had the higher per-core CDR than TP FUS-TB (30% vs. 55%). On the other hand, sensitivity, specificity, positive predictive value, negative predictive value, and diagnostic accuracy were equal between TP and TR approaches.

Stabile et al. compared the rate of detection of csPCa of different MRI TB approaches in their comparative, single-center study of patients undergoing both SB and FUS-TB or COG-TB at [19]. There were three operators, each one exclusively performing either COG-TB, TR FUS-TB, or TP FUS-TB. On multivariable analysis for patients undergoing FUS-TB, the TP approach was an independent predictor of csPCa detection (OR 4.1, 95% CI 1.4–12.9; *p* = 0.01). However, given the methodology, it is difficult to determine whether the differences were technique-specific or operator-specific.

Recently, Winoker et al. compared patients undergoing either TP or TR FUS-TB in a prospective non-randomized single institution series [20]. The CDR of csPCa by TP and TR FUS-TB were 59% and 54%, respectively. On multivariate analysis, there was no significant difference in detection of any PCa (OR 0.98, 95% CI 0.56–1.71; *p* = 0.940) or csPCa (OR 0.94, 95% CI 0.58–1.51; *p* = 0.791). On subgroup analysis with the PIRADS score, lesion volume, and location, FUS-TB was found to detect higher GG than SB for both TP and TR approaches. Neither TP nor TR cohorts showed any serious (Clavien–Dindo classification ≥ 3) complications.

Borkowetz et al. performed a prospective evaluation of TP FUS-TB compared to SB [21]. They evaluated 214 biopsy-naïve men, performing both TB (median 6 cores) and SB on patients with PIRADS scores ≥ 2. In their cohort, rates of diagnosis of csPCa (GG ≥ 2) were not significantly different between TB (38%) and SB (35%). However, they found a significantly higher CDR for combined SB + TB (44%) compared to both modalities alone (*p* < 0.005 for both). They determined that TB alone would miss 14% of csPCa cases, compared to 21% for SB. The authors discuss that the equivalent CDR for TB and SB may be due to the small sample size or the biopsy-naïve nature of the population.

Exterkate et al. evaluated a combination of TP and TR biopsy on 665 men with prior negative SB [22]. The 234 men with PIRADS ≥ 3 lesions were randomized to either TP FUS-TB, TR COG-TB, or TR IB-TB, with a minimum of 2 cores per lesion. Only patients undergoing FUS-TB and COG-TB underwent concomitant SB. However, the majority of the analysis focuses on TB vs. SB, rather than comparing different targeted approaches. TB was found to detect a significantly higher percentage of csPCa compared to SB (34% vs. 16%, *p* < 0.001), while combined SB + TB detected 35% csPCa. TB detected csCPa (GG ≥ 2) in 13% of patients with negative SB, whereas SB detected csPCa in 1.3% of patients with negative TB. There was no significant difference between biopsy approach (TP vs. TR).

In summary, TP and TR approaches seem to provide a similar csPCa detection rate. However, there is a lack of multicenter prospective randomized clinical trials comparing TP vs. TR SB and FUS-TB approaches in a large population.

## 5. Cognitive Fusion Versus Software-Guided Fusion-Targeted Biopsy

One of the first and largest studies to compare various TB techniques was performed by Delongchamps et al., in which 391 consecutive patients underwent SB followed by TB. The first 127 patients underwent COG-TB, the next 131 underwent rigid FUS-TB, and the final 133 underwent elastic FUS-TB. The overall CDR was higher with both FUS-TB methods than with SB, although COG-TB did not outperform SB [23]. Puech et al. then sought to compare the detection of csPCa in a prospective multicenter study of 95 patients undergoing 12-core SB, along with a 4-core TB (2 cores COG-TB, and 2 cores FUS-TB) [24]. Overall, CDR was 59% for SB and 69% for TB, while csPCa was diagnosed in 52% of SB compared to 67% of TB. There was no significant difference in CDR between COG-TB and FUS-TB. This study was followed by several other groups comparing COG-TB, FUS-TB, and SB. In one retrospective study evaluating the TR approach, 150 patients underwent COG-TB, 81 underwent FUS-TB, and 100 underwent SB. FUS-TB had the highest overall CDR compared to COG-TB and SB. TB had higher detection rates of GG ≥ 2, and lower rates of GG1 detection, compared to SB, but there was no significant difference between FUS-TB and COG-TB [25]. Valerio et al. reported a prospective study of 50 men, all of whom underwent FUS-TB, then COG-TB, then templated TP prostate biopsy in consecutive fashion [26]. The median number of cores taken was 3, 4, and 32, respectively. The CDR was 64%, 68%, and 76%, respectively, although combining the TB samples led to a combined CDR of 78%. Lastly, there was a trend towards an increase in detection of csPCa in the FUS-TB samples, although this did not reach statistical significance (*p* = 0.48).

Multiple groups directly compared COG-TB to FUS-TB, with reported data supporting a higher overall CDR, especially in smaller lesions, with FUS-TB [27,28]. A systematic review and meta-analysis of all studies comparing COG-TB and FUS-TB demonstrated a signal towards improved CDR with FUS-TB compared to COG-TB that did not reach statistical significance (*p* = 0.30) [29].

The PROFUS trial is a prospective trial comparing different TB methods in the same patient [1]. First, 125 patients with suspicious lesions on mpMRI underwent 2-core FUS-TB, followed by target blinding and 2-core COG-TB and 12-core SB by a second operator. When comparing pooled TB (FUS-TB + COG-TB) to SB, TB and SB had equivalent rates of GG ≥ 2 PCa (both 32.8%), although SB detected a significantly higher proportion of GG1 disease (*p* = 0.0044). They reported no cases of GG ≥ 2 PCa detected by the addition of SB to TB. In their analysis of FUS-TB vs. COG-TB, overall CDR for FUS-TB and COG-TB were 32.0% and 26.7%, respectively (*p* = 0.1374). Detection of GG ≥ 2 PCa was higher on FUS-TB (20.3%) compared to COG-TB (15.1%), *p* = 0.0523. A multivariate analysis demonstrated that the diameter of the suspicious lesion was significantly associated with CDR on FUS-TB, while smaller prostates and higher suspicion scores (4 and 5) were associated with increased CDR on all TB (FUS-TB + COG-TB). This trial is one of the first of its kind to allow for direct comparison of FUS-TB and COG-TB in the same patient. These studies suggest that TB has similar rates of csPCa detection compared to SB, while reducing detection of ciPCa. They also suggest that FUS-TB may detect higher rates of csCPa compared to COG-TB, with results approaching significance.

More recently, the PAIREDCAP trial prospectively evaluated 300 biopsy-naïve men with elevated prostate-specific antigen (PSA) or abnormal digital rectal exam (DRE), who underwent mpMRI [30]. Patients with PIRADS ≥ 3 underwent 12-core SB, as well as 3 cores each of both COG-TB and FUS-TB. The overall csPCa detection rate in men with MRI-visible lesions for all methods combined was 70.2%. CDR on a per-core basis for csPCa (GG ≥ 2) was 16% for SB, 33% for COG-TB, and 38% for FUS-TB. Combined COG-TB and FUS-TB missed 20.9% of patients with any PCa detected by SB. Conversely, 9.7% of men had any PCa detected by TB and missed on SB. In comparing concordance of COG-TB vs. FUS-TB, they found that csPCa was exclusively detected by FUS-TB in 24.7% of patients, and by COG-TB in 13.0% of patients, as well as by both COG-TB and FUS-TB in 64.2% of patients. The authors note the substantial discordance between TB and SB, postulating that these biopsy methods may target a different subset of tumors. Given the 15% csPCa CDR in the MRI-negative patient who underwent SB alone, the authors recommend continued use of SB along with TB. Both the PROFUS and the PAIREDCAP trials reported increased rates of PCa detection in FUS-TB compared to COG-TB, although the significance of these findings is yet to be determined.

Given the relative novelty of TB, the learning curve may also be a confounding factor in these initial comparison studies. Stabile et al. analyzed 244 patients who underwent a TRUS SB, and either COG-TB or FUS-TB [19], finding a significantly higher rate of overall and csPCa detection in the FUS-TB group. However, on multivariate analysis, operator expertise was independently associated with increased cancer detection, regardless of the fusion method used. Khoo et al. then reported on 1841 patients undergoing TP biopsy [31]. The overall csPCa detection rate was similar between COG-TB and FUS-TB, but with senior operators, there was a higher csPCa detection rate with FUS-TB.

Overall, while COG-TB does not require extra equipment, it is of paramount importance that operators have sufficient expertise in the technique. Although CDR may be similar for COG-TB and FUS-TB, FUS-TB performs better for smaller lesions and provides greater histologic information that may impact treatment decision. Additionally, FUS-TB may reduce learning curve and expertise dependency [1].

## 6. In-Bore Versus Cognitive Fusion Versus Software-Guided Fusion-Targeted Biopsy

The initial prospective study of MRI-guided IB-TB was reported by Quentin et al. and included 128 biopsy-naïve men [32]. The patients first underwent IB-TB, followed by SB. Overall and csPCa CDR were similar for both techniques. However, IB-TB required a lower number of cores while finding a higher percentage of cancer per core, indicating improved efficiency compared to SB. Another study retrospectively compared COG-TB + SB to IB-TB in biopsy-naïve patients, prior-negative biopsy patients, and patients on active surveillance [33]. Patients underwent either COG-TB + SB or IB-TB of PIRADS ≥ 3 (some PIRADS ≥ 2 with high clinical suspicion) lesions. In patients undergoing both TB and SB, GG ≥ 2 was diagnosed in 20/64 patients on COG-TB and 7/64 patients on SB. Of patients with negative COG-TB, no patients had GG ≥ 2 PCa on SB. Of those with negative SB, 13 patients had GG ≥ 2 PCa on COG-TB. Both COG-TB alone and IB-TB had equivalent proportions of GG ≥ 2 PCa (63%). When stratified by lesion size, IB-TB was found to detect a significantly higher proportion of PCa in smaller lesions (0–1.5 mL) compared to COG-TB: 69% and 39%, respectively, *p* = 0.009, which may support a role for IB-TB in smaller lesions. This study is valuable in that it evaluates different TB modalities; however, it is limited by the patient population, comparing COG-TB + SB to an already collected retrospective cohort of IB-TB patients.

The difference in csPCa detection between FUS-TB and IB-TB was explored in a retrospective analysis, demonstrating no significant difference in csPCa detection rate with 49% in the FUS-TB group and 61% in the IB-TB group. These results are in contrast to a later study which also compared csPCa detection between 300 FUS-TB and 103 IB-TB patients [34], which found that IB-TB led to a higher rate of csPCa detection and lower rate of ciPCa detection, as well as less frequent GG upgrading on prostatectomy. Another prospective trial randomized 210 men with at least one prior negative prostate biopsy. After mpMRI, patients were randomized to either IB-TB or FUS-TB and SB. The study was closed prematurely after the primary endpoint of an overall CDR of at least 60% in the FUS-TB and SB group was not met in an interim analysis. The overall CDR was 37% in the IB-TB group and 39% in the FUS-TB and SB group. There were no statistically significant differences in csPCa detection rates between the two groups [35].

The FUTURE trial was a multicenter randomized controlled trial involving 665 patients with prior negative SB [36]. Participants underwent TB if a PIRADS ≥ 3 lesion was identified on mpMRI, and were randomized in a 1:1:1 fashion to FUS-TB, COG-TB, or IB-TB. In total, 234 patients ultimately underwent TB, and there were no statistically significant differences in the overall (49% vs. 44% vs. 55%, respectively; *p* = 0.4) or csPCa 34%, 33%, and 33%, respectively; *p* > 0.9) CDR between the three methods. The authors did note a 50% lower power than anticipated due to the lower yield of PIRADS ≥ 3 lesions on mpMRI. A larger trial would be of benefit, but a post hoc power analysis indicated that 9886 patients would be required with the same trial design.

While IB-TB might have the potential to be the most precise target strategy as it does not require image fusion and lesions are directly targeted on MRI, the CDR is similar to other fusion strategies. IB-TB is costly and time-consuming, requiring an MRI suite and MRI-compatible equipment and supplies, as well as the expertise to use them. Approximately 15% of csPCa are invisible on MRI, and therefore will not be detected on IB-TB [2]. In addition, SB, which may improve CDR when combined with TB, cannot be performed during IB-TB. Given these limitations, some authors advocate the use of IB-TB specifically for very small lesions, and/or repeat biopsy when clinical suspicion is high but prior TB are negative [37].

## 7. Alternative mpMRI Protocols

As the evidence supporting mpMRI accumulates, there has been discussion regarding the ability of MRI to act as a screening test. Although traditional mpMRI is more time-consuming and expensive than TRUS and PSA, there have been several recent efforts to optimize prostate MRI efficiency.

Biparametric MRI (bpMRI) utilizes only the T2 and the diffusion-weighted imaging (DWI) or apparent diffusion coefficient (ADC) phases. The dynamic contrast-enhanced (DCE) phase plays a predominant role in post-treatment evaluation [38]. A recent prospective, blinded-cohort study evaluated bpMRI combined with ultrasonography (B-mode and shear wave elastography) as an alternative to population screening for PCa [39]. Any suspicious lesions identified on mpMRI (internal mpMRI score 3–5) or TRUS were targeted in the biopsy, in addition to a 12-core TP biopsy. Of the 406 patients, 17 were found to have csPCa, of which bpMRI identified 14, ultrasound detected 9, and PSA detected 7 cases. Alabousi et al., in their systematic review and meta-analysis, determined that there was no significant difference in diagnostic test accuracy between mpMRI and bpMRI in diagnosing PCa in treatment-naïve patients [38]. However, even bpMRI may be able to undergo further optimization. Van der Leest et al. evaluated the performance of “fast” bpMRI (monopolar) as a “rule out” test for high-risk PCa [40]. They compared fast bpMRI to triplanar bpMRI and standard mpMRI, with a negative predictive value (NPV) for high-grade PCa of 97% for all three modalities (fast bpMRI had a significantly lower NPV for high-grade PCa by 0.15%, *p* < 0.001). They determined that using the fast bpMRI technique, they could double their capacity for prostate MRI while reducing costs.

Another alternative to improve MRI efficiency is the “one-stop” pathway, in which mpMRI and MRI-TB (SB if MRI-negative) are performed on the same day, reducing time to diagnosis and decreasing the financial and time burden to the patient. Tafuri et al. found that the one-stop pathway had similar csPCa detection rate when compared to a traditional pathway (49% one-stop vs. 41% traditional, *p* = 0.55) [41].

Fast, rapid, and one-stop MRI protocols, as well as AI and automatization of MRI reading, would likely increase cost-effectiveness, decrease operator dependency, and improve interobserver variability. This may allow for MRI screening for a populational-based setting, similar to low-dose CT for lung cancer [42].

We summarized high impact manuscripts reviewed in this manuscript in the Table 1.

## 8. Conclusions

TB has been demonstrated to provide a significant diagnostic advantage when combined with SB. There is a growing subset of data which supports the role of TB alone, which may allow for increased efficiency and decreased complications. Although there are limited data which directly compare different TB modalities, as well as TP vs. TR approaches, we presented the existing, high-quality analyses on these subjects.

**Table 1:** Summary of high-impact manuscripts reviewed in this manuscript.

Author Year	Study Design	Patient Population	Definition of csPCa	Comparison	Endpoint	Outcomes	Limitations
**Systematic versus Targeted Biopsy**							
Kasivisvanathan et al. (PRECISION) [3] 2018	Prospective, multicenter, randomized controlled, noninferiority trial	500 patients, biopsy-naïve	GG ≥ 2	MRI pathway (TB without SB if the MRI was suggestive of PCa, no Bx if the MRI was not suggestive of PCa) vs. 10–12 SB	Proportion of men who received a diagnosis of clinically significant cancer	csPCa CDR for MRI pathway vs. SB were 38% vs. 26% (*p* = 0.005). MRI pathway showed both noninferiority and superiority. ciPCa CDR for MRI pathway (9%) was significantly lower than SB (22%) (*p* < 0.001)	Moderate agreement (78%) among the sites and the radiologists reporting. MRI invisible csPCa can be missed on MRI pathway group
Ahdoot et al. [4] (Trio) 2020	Large, single-center, prospective, clinical trial	2103 patients with elevated PSA or abnormal DRE and MRI suspicious lesion for PCa	GG ≥ 3	FUS-TB vs. SB vs. TB + SB	Cancer detection according to GG	CDR on FUS-TB was significantly lower for GG 1 PCa and higher for GG ≥ 3 PCa. CDRs for GG ≥ 2 PCa were 31% with SB and 37.8% with TB. TB alone missed GG ≥ 2 PCa in 5.8% of patients and GG ≥ 3 in 1.9% of patients. Rates of upgrading on prostatectomy specimens were significantly higher for SB 41.6% (16.8% upgrading to GG ≥ 3) compared to TB 30.9% (8.7% upgrading to GG ≥ 3)	FUS-TB performed before SB may have affected the performance of SB. A single-center study may lead to limited generalizability
Filson et al. [7] 2016	Large, single-center, prospective, clinical trial	1042 patients with elevated PSA or abnormal digital rectal examination or considering confirmation biopsy for active surveillance	GG ≥ 2	FUS-TB vs. SB vs. TB + SB	csPCa detection	csPCa CDRs of TB alone vs. SB alone vs. TB + SB were 28% vs. 24% vs. 35%, respectively. TB + SB detected a significantly higher proportion of csPCa compared to both SB and TB alone (*p* < 0.001 for both).	The MRI scoring system in this study was institution-specific, although the protocol was similar to PIRADS
**Systematic versus Targeted Biopsy in the Biopsy-Naïve Setting**							
Pokorny et al. [9] 2014	Single-center, prospective study	223 patients, biopsy-naïve	None	SB vs. TB vs. SB + TB	PCa detection	Overall CDR for SB was 56.5%, with 62.7% intermediate/high risk PCa (high volume GG2 or GG ≥ 3), compared to CDR 69.7% with 93.9% intermediate/high risk PCa for TB. In combined SB + TB, CDR was 64%, of which 76% were intermediate/high risk PCa.	Lack of oncologic follow-up data. Combination of TB and SB (TB first) can affect SB CDR
Van der Leest et al. [10] (4M) 2019	Multicenter, prospective study	626 patients, biopsy-naïve	GG ≥ 2	IB-TB vs. SB MRI pathway (patients with PIRADS ≥ 3 lesions underwent IB-TB followed by SB) vs. TRUSGB pathway (MRI-negative patients underwent SB alone)	The overall detection rates of csPCa and ciPCa for both pathways	IB-TB detected csPCa in 50% of patients with PIRADS ≥ 3 lesions, while combination IB-TB and SB detected csPCa in 57%. Overall, the TRUSGB pathway identified csPCa in 23%, compared to 25% in the MRI pathway. IB-TB alone underdetected 9% of csCPa vs. 2% in the combined approach. The sensitivity of the combined approach vs. SB was not significantly different in the detection of csPCa.	Combination of TB and SB (TB first) can affect SB CDR
Rouviere et al. [11] (MRI-FIRST) 2019	Multicenter, prospective, paired diagnostic study	224 patients, biopsy-naïve	GG ≥ 2 (csPCa-A), GG 1 with MCCL ≥ 6 mm or GG ≥ 2 (csPCa-B), GG ≥ 3 (csPCa-C)	TB (COG-TB or FUS-TB) vs. SB	Detection of csPCa-A	GG ≥ 2 PCa was diagnosed in 32.3% of TB alone, 29.9% of SB alone, and 37.5% of combined PBx. SB and TB CDRs of GG ≥ 2 PCa were not significantly different. TB detected a significantly higher rate of GG ≥ 3 PCa (19.9% vs. 15.1%, *p* = 0.0095). SB detected a significantly higher proportion of ciPCa (20% vs. 5.8%, *p* < 0.0001). SB alone would miss 7.6% of csPCa, while mpMRI would miss 5.2%.	Combination of TB and SB (TB first) can affect SB CDR
Klotz et al. (12) 2021	Prospective, multicenter, randomized control, noninferiority trial	453 patients, biopsy-naïve	GG ≥ 2	MRI pathway with 4 cores per lesion vs. 12 core SB	Proportion of men with csPCa diagnosed in each arm	csPCa CDR for SB group vs. MRI pathway group were 30% vs. 35% (absolute difference, 5%, 97.5% 1-sided CI, −3.4% to ∞; noninferiority margin, −5%). The superiority test deemed not significant (*p* = 0.54). ciPCa CDR were significantly lower in the MRI pathway arm (10.1% vs. 21.7%; absolute difference, 11.6%; 95% CI, −18.2% to −4.9%; *p* < 0.001).	MRI invisible csPCa can be missed on MRI pathway group
**Systematic versus Targeted Biopsy Using the Transperineal Approach**							
Pepe et al. [18] 2017	Prospective study	150 patients with PBx history (repeat PBx)	GG ≥ 2 and/or more than 2 positive core	TP vs. TR FUS-TB	Detection rate for csPCa with TP vs. TR FUS-TB	The detection rate for csPCa using TP FUS-TB was higher than TR (89.1% vs. 78.1%). The CDR in the anterior zone for TP approach was higher than TR approach (86.7% vs. 46.7%; *p* = 0.0001).	Sequential TP, TR FUS-TB, and saturation PBx can affect accuracy of biopsy
Winoker et al. [20] 2020	Prospective study	379 patients at risk of PCa and with an MRI visible lesion	GG ≥ 2	TP vs. TR FUS-TB	PCa detection of men with MRI visible lesions	The CDR of csPCa by TP and TR FUS-TB were 59% and 54% (*p* = 0.3), respectively. On multivariate analysis, there was no significant difference in detection of any PCa (OR 0.98, 95% CI 0.56–1.71; *p* = 0.940) or csPCa (OR 0.94, 95% CI 0.58–1.51; *p* = 0.791). There were no serious (Clavien ≥ 3) complications following PBx in both approach method.	Nonrandomized selection may lead to bias
Borkowetz et al. [21] 2018	Prospective, multicenter trial	214 patients, biopsy-naïve	GG ≥ 2	TP FUS-TB vs. TR SB	Proportion of patients diagnosed with significant PCa	csPCa CDRs were not significantly different between TP FUS-TB (38%) and TR SB (35%). CDR for combined SB + TB (44%) were significantly higher than both modalities alone (*p* < 0.005 for both).	Same operator performed TB and SB without being blinded to the cancer suspicious lesion on mpMRI, which may have impacted the performance of SB. Unlike other studies, FUS-TB was performed for PIRADS ≥ 2 lesions
Exterkate et al. [22] (FUTURE) 2020	Prospective, multicenter, randomized controled trial	152 patients with PIRADS ≥ 3 and prior negative SB. They underwent TP FUS-TB or TR COG-TB in combination with SB	GG ≥ 2	TB vs. SB	Detection difference between TB and repeated SB (secondary endpoint)	csPCa CDR for TB vs. SB were 32% vs. 16% (*p* < 0.001). Compared with TB alone, combination of TB and SB resulted in CDR differences of 1.0%, 5.0%, and 6.0% for csPCa, ciPCa, and any PCa, respectively. There was no significant difference between biopsy approach (TP vs. TR).	This trial was designed to compare CDRs of three TB techniques; therefore, sample size calculations for subgroup analyses are lacking. Same operator performed TB and SB without being blinded to the cancer suspicious lesion on mpMRI
**Cognitive Fusion versus Software-Guided Fusion Targeted Biopsy**							
Delongchamps et al. [23] 2013	Prospective study	391 patients, biopsy-naïve	GG ≥ 2	COG-TB vs. rigid FUS-TB vs. elastic FUS-TB	The accuracy of COG-TB vs. rigid FUS-TB vs. elastic FUS-TB	The overall CDR was 42%, 59%, and 62% with COG-TB, rigid FUS-TB, and elastic FUS-TB, respectively. The CDRs were significantly higher with both FUS-TB methods than with SB, while COG-TB did not outperform SB.	Internal scoring system for MRI. Study was not randomized
Puech et al. [24] 2013	Prospective, multicenter study	95 patients with PCa suspicious lesion on MRI	Any ≥ 3 mm core cancer length or any GG ≥ 2 for SB or any cancer length for TB	SB vs. COG-TB vs. FUS-TB	Core cancer length	Overall CDR was 59% for SB and 69% for TB. csPCa CDR was in 52% of SB compared to 67% of TB. There was no significant difference in CDR between COG-TB and FUS-TB.	Internal scoring system for MRI. Sequential SB and TB can affect TB performance
Wysock et al. [1] (PROFUS) 2014	Prospective study	125 patients with PCa suspicious lesion on MRI	>5 mm total cancer length and/or any GG ≥ 2	FUS-TB vs. COG-TB vs. SB		Pooled TB (FUS-TB + COG-TB) and SB had equivalent rates of GG ≥ 2 PCa (both 32.8%), while SB detected a significantly higher proportion of GG1 disease (*p* = 0.0044). Overall CDR for FUS-TB and COG-TB was 32.0% and 26.7%, respectively (*p* = 0.1374). Detection of GG ≥ 2 PCa was higher on FUS-TB (20.3%) compared to COG-TB (15.1%), (*p* = 0.0523). A multivariate analysis demonstrated that the diameter of the suspicious lesion was significantly associated with CDR on FUS-TB. Smaller prostates and higher suspicion scores were associated with increased CDR on all TB (FUS-TB + COG-TB).	This study was not powered to compare several TB methods and SB. Sequential TB and SB can affect SB performance
Elkhoury et al. [30] (PAIREDCAP) 2019	Prospective, single-center, paired cohort trial	300 patients, biopsy-naïve	GG ≥ 2	SB vs. COG-TB vs. FUS-TB	Detection of clinically significant cancer	The overall csPCa detection rate in men with PIRADS ≥ 3 lesion for all methods’ combination was 70.2%. CDR on a per-core basis for csPCa was 16% for SB, 33% for COG-TB, and 38% for FUS-TB. The combination of COG-TB and FUS-TB missed 20.9% of patients with any PCa detected by SB, while 9.7% of men had any PCa detected by TB and missed on SB. In comparing concordance of COG-TB vs. FUS-TB, csPCa was exclusively detected by FUS-TB in 24.7% of patients, and by COG-TB in 13.0% of patients, as well as by both COG-TB and FUS-TB in 64.2% of patients.	Single-center study; single operator
**In-Bore versus Cognitive Fusion versus Software-Guided Fusion Targeted Biopsy**							
Arsov et al. [35] 2015	Prospective, single-center, randomized controlled trial	210 patients with at least one negative TRUS-guided biopsy and persistent PSA levels ≥ 4 ng/mL	GG ≥ 2	IB-TB alone vs. FUS-TB + SB	Overall PCa detection rate	The PCa CDR was 37% in the IB-TB arm and 39% in the FUS-TB and SB arm (*p* = 0.7). There were no statistically significant differences in csPCa CDR between the two arms (29% vs. 32%, *p* = 0.7)	Single-center study. Only one type of fusion biopsy device. Combination of TB and SB (TB first) can affect SB CDR. The primary endpoint was not csPCa detection but an overall PCa detection. The endpoint was not met after interim analysis
Wegelin et al. [36] (FUTURE) 2019	Prospective, multicenter, randomized controlled trial	665 patients with prior negative SB	GG ≥ 2	FUS-TB vs. COG-TB vs. IB-TB (234 patients with PIRADS ≥ 3 were randomized to TP FUS-TB, TR COG-TB, and TR IB-TB)	Overall PCa detection	No statistically significant differences were observed in overall (49% vs. 44% vs. 55%, *p* = 0.4) or csPCa (34% vs. 33% vs. 33%, *p* > 0.9) CDR between the three methods	Underpowering for primary outcome (overall PCa detection) due to a low rate of PIRADS ≥ 3 lesions on mpMRI
**Accuracy of MRI Predicting Prostate Cancer**							
Ahmed et al. (PROMIS) [43] 2017	Multicenter, paired-cohort confirmatory study	576 patients, biopsy-naïve	GG ≥ 3 more, or MCCL ≥ 6 mm	mpMRI and 10–12 core SB vs. template mapping biopsy	Proportion of men who could safely avoid biopsy and proportion of men who had csPCa and were correctly identified by mpMRI	csPCa CDR was more sensitive with mpMRI than SB (93% vs. 48%, *p* < 0.0001) and less specific (41% vs. 96%, *p* < 0.0001)	Patients with prostate size > 100 mL were excluded due to template grid size and pubic arch interference. Template biopsy followed by SB may have decreased accuracy due to prostate swelling or deformation
Simmons et al. (PICTURE) [44] 2017	Single-center, paired- cohort study	249 men with prior biopsy	GG ≥ 3 more and/or cancer core length ≥ 6 mm	mpMRI with template mapping biopsy as reference test	Number of men who could avoid repeat PBx by mpMRI for csPCa	The accuracy assessed by AUROC/sensitivity/specificity of mpMRI with Likert score ≥ 3 cutoff were 0.74%/97.1%/21.9%. A total of 35/249 of men with scores < 3 could potentially avoid biopsy, with 32/35 patients with ciPCa or benign disease, and 3/35 patients with csPCa that would be missed	Low proportion of patients with Likert score 1 or 2 (14%) may lead to low specificity. The study was conducted before PIRADS era

GG, Gleason grade; mpMRI, multiparametric magnetic resonance imaging; PBx, prostate biopsy; SB, systematic biopsy; PIRADS, Prostate Imaging Reporting and Data System; MCCL, maximum cancer core length; PCa, prostate cancer; csPCa, clinically significant prostate cancer; ciPCa, clinically insignificant prostate cancer; MRI-TB, MRI-targeted biopsy; FUS-TB, software fusion-targeted biopsy; IB-TB, in-bore targeted biopsy; CDR, cancer detection rate; TP, transperineal; TR, transrectal; DRE, digital rectal exam.

## Data Availability

No new data were created or analyzed in this study. Data sharing is not applicable to this article.

## References

[1] Wysock J.S., Rosenkrantz A.B., Huang W.C., Stifelman M.D., Lepor H., Deng F.-M., Melamed J., Taneja S.S. (2014). A Prospective, Blinded Comparison of Magnetic Resonance (MR) Imaging–Ultrasound Fusion and Visual Estimation in the Performance of MR-targeted Prostate Biopsy: The PROFUS Trial. Eur. Urol..

[2] Oishi M., Shin T., Ohe C., Nassiri N., Palmer S.L., Aron M., Ashrafi A.N., Cacciamani G.E., Chen F., Duddalwar V. (2019). Which Patients with Negative Magnetic Resonance Imaging Can Safely Avoid Biopsy for Prostate Cancer?. J. Urol..

[3] Kasivisvanathan V., Rannikko A.S., Borghi M., Panebianco V., Mynderse L.A., Vaarala M.H., Briganti A., Budäus L., Hellawell G., Hindley R.G. (2018). MRI-Targeted or Standard Biopsy for Prostate-Cancer Diagnosis. N. Engl. J. Med..

[4] Ahdoot M., Wilbur A.R., Reese S.E., Lebastchi A.H., Mehralivand S., Gomella P.T., Bloom J., Gurram S., Siddiqui M., Pinsky P. (2020). MRI-Targeted, Systematic, and Combined Biopsy for Prostate Cancer Diagnosis. N. Engl. J. Med..

[5] Siddiqui M.M., Rais-Bahrami S., Turkbey B., George A.K., Rothwax J., Shakir N., Okoro C., Raskolnikov D., Parnes H.L., Linehan W.M. (2015). Comparison of MR/Ultrasound Fusion–Guided Biopsy With Ultrasound-Guided Biopsy for the Diagnosis of Prostate Cancer. JAMA.

[6] Calio B.P., Sidana A., Sugano D., Gaur S., Maruf M., Jain A.L., Merino M.J., Choyke P.L., Wood B.J., Pinto P.A. (2018). Risk of Upgrading from Prostate Biopsy to Radical Prostatectomy Pathology—Does Saturation Biopsy of Index Lesion during Multiparametric Magnetic Resonance Imaging-Transrectal Ultrasound Fusion Biopsy Help?. J. Urol..

[7] Filson C.P., Natarajan S., Margolis D.J., Huang J., Lieu P., Dorey F.J., Reiter R.E., Marks L.S. (2016). Prostate cancer detection with magnetic resonance-ultrasound fusion biopsy: The role of systematic and targeted biopsies. Cancer.

[8] Maxeiner A., Kittner B., Blobel C., Wiemer L., Hofbauer S.L., Fischer T., Asbach P., Haas M., Penzkofer T., Fuller F. (2018). Primary magnetic resonance imaging/ultrasonography fusion-guided biopsy of the prostate. BJU Int..

[9] Pokorny M.R., de Rooij M., Duncan E., Schröder F.H., Parkinson R., Barentsz J.O., Thompson L.C. (2014). Prospective Study of Diagnostic Accuracy Comparing Prostate Cancer Detection by Transrectal Ultrasound–Guided Biopsy Versus Magnetic Resonance (MR) Imaging with Subsequent MR-guided Biopsy in Men Without Previous Prostate Biopsies. Eur. Urol..

[10] Van der Leest M., Cornel E., Israël B., Hendriks R., Padhani A.R., Hoogenboom M., Zamecnik P., Bakker D., Setiasti A.Y., Veltman J. (2019). Head-to-head Comparison of Transrectal Ultrasound-guided Prostate Biopsy Versus Multiparametric Prostate Resonance Imaging with Subsequent Magnetic Resonance-guided Biopsy in Biopsy-naïve Men with Elevated Prostate-specific Antigen: A Large Prospective Multicenter Clinical Study. Eur. Urol..

[11] Rouvière O., Puech P., Renard-Penna R., Claudon M., Roy C., Mège-Lechevallier F., Decaussin-Petrucci M., Dubreuil-Chambardel M., Magaud L., Remontet L. (2019). Use of prostate systematic and targeted biopsy on the basis of multiparametric MRI in biopsy-naive patients (MRI-FIRST): A prospective, multicentre, paired diagnostic study. Lancet Oncol..

[12] Klotz L., Chin J., Black P.C., Finelli A., Anidjar M., Bladou F., Machado A., Levental M., Ghai S., Chang S. (2021). Comparison of Multiparametric Magnetic Resonance Imaging–Targeted Biopsy With Systematic Transrectal Ultrasonography Biopsy for Biopsy-Naive Men at Risk for Prostate Cancer. JAMA Oncol..

[13] Kenigsberg A.P., Llukani E., Deng F.-M., Melamed J., Zhou M., Lepor H. (2018). The Use of Magnetic Resonance Imaging to Predict Oncological Control Among Candidates for Focal Ablation of Prostate Cancer. Urolology.

[14] Borghesi M., Ahmed H., Nam R., Schaeffer E., Schiavina R., Taneja S., Weidner W., Loeb S. (2017). Complications After Systematic, Random, and Image-guided Prostate Biopsy. Eur. Urol..

[15] Barnett C.L., Davenport M.S., Montgomery J.S., Wei J.T., Montie J.E., Denton B.T. (2018). Cost-effectiveness of magnetic resonance imaging and targeted fusion biopsy for early detection of prostate cancer. BJU Int..

[16] Loy L.M., Lim G.H., Leow J.J., Lee C.H., Tan T.W., Tan C.H. (2020). A systematic review and meta-analysis of magnetic resonance imaging and ultrasound guided fusion biopsy of prostate for cancer detection—Comparing transrectal with transperineal approaches. Urol. Oncol. Semin. Orig. Investig..

[17] Xiang J., Yan H., Li J., Wang X., Chen H., Zheng X. (2019). Transperineal versus transrectal prostate biopsy in the diagnosis of prostate cancer: A systematic review and meta-analysis. World J. Surg. Oncol..

[18] Pepe P., Garufi A., Priolo G.D., Pennisi M. (2017). Multiparametric MRI/TRUS Fusion Prostate Biopsy: Advantages of a Transperineal Approach. Anticancer Res..

[19] Stabile A., Dell’Oglio P., Gandaglia G., Fossati N., Brembilla G., Cristel G., Dehò F., Scattoni V., Maga T., Losa A. (2018). Not All Multiparametric Magnetic Resonance Imaging–targeted Biopsies Are Equal: The Impact of the Type of Approach and Operator Expertise on the Detection of Clinically Significant Prostate Cancer. Eur. Urol. Oncol..

[20] Winoker J.S., Wajswol E., Falagario U., Maritini A., Moshier E., Voutsinas N., Knauer C.J., Sfakianos J.P., Lewis S.C., Taouli B.A. (2020). Transperineal Versus Transrectal Targeted Biopsy With Use of Electromagnetically-tracked MR/US Fusion Guidance Platform for the Detection of Clinically Significant Prostate Cancer. Urology.

[21] Borkowetz A., Hadaschik B., Platzek I., Toma M., Torsev G., Renner T., Herout R., Baunacke M., Laniado M., Baretton G. (2018). Prospective comparison of transperineal magnetic resonance imaging/ultrasonography fusion biopsy and transrectal systematic biopsy in biopsy-naïve patients. BJU Int..

[22] Exterkate L., Wegelin O., Barentsz J.O., Van Der Leest M.G., Kummer J.A., Vreuls W., De Bruin P.C., Bosch J.R., Van Melick H.H., Somford D.M. (2020). Is There Still a Need for Repeated Systematic Biopsies in Patients with Previous Negative Biopsies in the Era of Magnetic Resonance Imaging-targeted Biopsies of the Prostate?. Eur. Urol. Oncol..

[23] Delongchamps N.B., Peyromaure M., Schull A., Beuvon F., Bouazza N., Flam T., Zerbib M., Muradyan N., Legman P., Cornud F. (2013). Prebiopsy Magnetic Resonance Imaging and Prostate Cancer Detection: Comparison of Random and Targeted Biopsies. J. Urol..

[24] Puech P., Rouviere O., Renard-Penna R., Villers A., Devos P., Colombel M., Bitker M.-O., Leroy X., Mège-Lechevallier F., Compérat E. (2013). Prostate Cancer Diagnosis: Multiparametric MR-targeted Biopsy with Cognitive and Transrectal US–MR Fusion Guidance versus Systematic Biopsy—Prospective Multicenter Study. Radiolology.

[25] Oberlin D.T., Casalino D.D., Miller F.H., Matulewicz R.S., Perry K.T., Nadler R.B., Kundu S., Catalona W.J., Meeks J.J. (2016). Diagnostic Value of Guided Biopsies: Fusion and Cognitive-registration Magnetic Resonance Imaging Versus Conventional Ultrasound Biopsy of the Prostate. Urology.

[26] Valerio M., McCartan N., Freeman A., Punwani S., Emberton M., Ahmed H.U. (2015). Visually directed vs. software-based targeted biopsy compared to transperineal template mapping biopsy in the detection of clinically significant prostate cancer. Urol. Oncol. Semin. Orig. Investig..

[27] Oderda M., Faletti R., Battisti G., Dalmasso E., Falcone M., Marra G., Palazzetti A., Zitella A., Bergamasco L., Gandini G. (2016). Prostate Cancer Detection Rate with Koelis Fusion Biopsies versus Cognitive Biopsies: A Comparative Study. Urol. Int..

[28] Yamada Y., Shiraishi T., Ueno A., Ueda T., Fujihara A., Naitoh Y., Hongo F., Ukimura O. (2020). Magnetic resonance imaging-guided targeted prostate biopsy: Comparison between computer-software-based fusion versus cognitive fusion technique in biopsy-naïve patients. Int. J. Urol..

[29] Watts K.L., Frechette L., Muller B., Ilinksy D., Kovac E., Sankin A., Aboumohamed A. (2020). Systematic review and meta-analysis comparing cognitive vs. image-guided fusion prostate biopsy for the detection of prostate cancer. Urol. Oncol. Semin. Orig. Investig..

[30] ElKhoury F.F., Felker E.R., Kwan L., Sisk A.E., Delfin M., Natarajan S., Marks L.S. (2019). Comparison of Targeted vs. Systematic Prostate Biopsy in Men Who Are Biopsy Naive. JAMA Surg..

[31] Khoo C.C., Eldred-Evans D., Peters M., van Son M., van Rossum P.S.N., Connor M.J., Hosking-Jervis F., Tanaka M.B., Reddy D., Bass E. (2021). A Comparison of Prostate Cancer Detection between Visual Estimation (Cognitive Registration) and Image Fusion (Software Registration) Targeted Transperineal Prostate Biopsy. J. Urol..

[32] Quentin M., Blondin D., Arsov C., Schimmöller L., Hiester A., Godehardt E., Albers P., Antoch G., Rabenalt R. (2014). Prospective Evaluation of Magnetic Resonance Imaging Guided In-bore Prostate Biopsy versus Systematic Transrectal Ultrasound Guided Prostate Biopsy in Biopsy Naïve Men with Elevated Prostate Specific Antigen. J. Urol..

[33] Osses D.F., Van Asten J.J., Tijsterman J.D. (2017). Cognitive-Targeted versus Magnetic Resonance Imaging-Guided Prostate Biopsy in Prostate Cancer Detection. Curr. Urol..

[34] Costa D.N., Goldberg K., De Leon A.D., Lotan Y., Xi Y., Aziz M., Freifeld Y., Margulis V., Raj G., Roehrborn C.G. (2019). Magnetic Resonance Imaging–guided In-bore and Magnetic Resonance Imaging-transrectal Ultrasound Fusion Targeted Prostate Biopsies: An Adjusted Comparison of Clinically Significant Prostate Cancer Detection Rate. Eur. Urol. Oncol..

[35] Arsov C., Rabenalt R., Blondin D., Quentin M., Hiester A., Godehardt E., Gabbert H.E., Becker N., Antoch G., Albers P. (2015). Prospective Randomized Trial Comparing Magnetic Resonance Imaging (MRI)-guided In-bore Biopsy to MRI-ultrasound Fusion and Transrectal Ultrasound-guided Prostate Biopsy in Patients with Prior Negative Biopsies. Eur. Urol..

[36] Wegelin O., Exterkate L., van der Leest M., Kummer J.A., Vreuls W., de Bruin P.C., Bosch J., Barentsz J.O., Somford D.M., van Melick H.H. (2019). The FUTURE Trial: A Multicenter Randomised Controlled Trial on Target Biopsy Techniques Based on Magnetic Resonance Imaging in the Diagnosis of Prostate Cancer in Patients with Prior Negative Biopsies. Eur. Urol..

[37] Venderink W., Bomers J.G., Overduin C.G., Padhani A.R., De Lauw G.R., Sedelaar M.J., Barentsz J.O. (2020). Multiparametric Magnetic Resonance Imaging for the Detection of Clinically Significant Prostate Cancer: What Urologists Need to Know. Part 3: Targeted Biopsy. Eur. Urol..

[38] Alabousi M., Salameh J.-P., Gusenbauer K., Samoilov L., Jafri A., Yu H., Alabousi A. (2019). Biparametric vs. multiparametric prostate magnetic resonance imaging for the detection of prostate cancer in treatment-naïve patients: A diagnostic test accuracy systematic review and meta-analysis. BJU Int..

[39] Eldred-Evans D., Burak P., Connor M.J., Day E., Evans M., Fiorentino F., Gammon M., Hosking-Jervis F., Klimowska-Nassar N., McGuire W. (2021). Population-Based Prostate Cancer Screening With Magnetic Resonance Imaging or Ultrasonography. JAMA Oncol..

[40] Van Der Leest M., Israël B., Cornel E.B., Zámecnik P., Schoots I.G., Van Der Lelij H., Padhani A.R., Rovers M., Van Oort I., Sedelaar M. (2019). High Diagnostic Performance of Short Magnetic Resonance Imaging Protocols for Prostate Cancer Detection in Biopsy-naïve Men: The Next Step in Magnetic Resonance Imaging Accessibility. Eur. Urol..

[41] Tafuri A., Ashrafi A.N., Palmer S., Shakir A., Cacciamani G.E., Iwata A., Iwata T., Cai J., Sali A., Gupta C. (2019). One-Stop MRI and MRI/transrectal ultrasound fusion-guided biopsy: An expedited pathway for prostate cancer diagnosis. World J. Urol..

[42] Varghese B., Chen F., Hwang D., Palmer S.L., Abreu A.L.D.C., Ukimura O., Aron M., Aron M., Gill I., Duddalwar V. (2019). Objective risk stratification of prostate cancer using machine learning and radiomics applied to multiparametric magnetic resonance images. Sci. Rep..

[43] Ahmed H.U., El-Shater Bosaily A., Brown L.C., Gabe R., Kaplan R., Parmar M.K., Collaco-Moraes Y., Ward K., Hindley R.G., Freeman A. (2017). Diagnostic accuracy of multi-parametric MRI and TRUS biopsy in prostate cancer (PROMIS): A paired validating confirmatory study. Lancet.

[44] Simmons L.A.M., Kanthabalan A., Arya M., Briggs T., Barratt D., Charman S.C., Freeman A., Gelister J., Hawkes D., Hu Y. (2017). The PICTURE study: Diagnostic accuracy of multiparametric MRI in men requiring a repeat prostate biopsy. Br. J. Cancer.

